# Daily Views on Aging, Positive and Negative Experiences – Findings From Diverse Contexts and Countries

**DOI:** 10.1093/geroni/igaf122.1489

**Published:** 2025-12-31

**Authors:** Anna Kornadt, Dwight Tse

## Abstract

Views on aging have recently been investigated regarding short term fluctuations and in relation to daily experiences. In the current symposium, we draw on international samples of the Subjective AGES consortium. We focus on how adverse and positive experiences are related to daily views on aging, such as subjective age and awareness of age-related changes. Senyk and colleagues investigate two samples of older adults from Ukraine and Israel find that war exposure increases subjective age. In a daily diary study with older adults from four different countries, Shrira et al. find that feeling closer to death on a given day decreases well-being, and that the strength of this relation was moderated by religious affiliation. Watson and Tse more broadly investigate the role of daily hassles and uplifts for subjective age among UK older adults. They find that especially uplifts contribute to a younger subjective age, potentially mediated by daily health problems. Finally, Wu and colleagues address the association between daily hassles and uplifts and views on aging among Hong Kong Chinese. Mattering and non-essential beliefs about aging were found to increase the positive impact of uplifts and attenuating the negative role of stressors on awareness of age-related changes. Taken together, our symposium shows that the study of daily life experiences and the mechanisms that relate them to views on aging enhance our understanding of psychological processes and dynamics among views on aging, contextual factors, and developmental outcomes.

